# The genetics of phenotypic plasticity. X. Variation versus uncertainty

**DOI:** 10.1002/ece3.217

**Published:** 2012-04

**Authors:** Samuel M Scheiner, Robert D Holt

**Affiliations:** 1 Division of Environmental Biology, National Science Foundation4201 Wilson Boulevard, Arlington, Virginia 22230; 2 Department of Biology, University of FloridaGainesville, Florida 32611

**Keywords:** Baldwin effect, bet hedging, genetic architecture, limitations, model, theory

## Abstract

Despite the apparent advantages of adaptive plasticity, it is not common. We examined the effects of variation and uncertainty on selection for plasticity using an individual-based computer simulation model. In the model, the environment consisted of a linear gradient of 50 demes with dispersal occurring either before or after selection. Individuals consisted of multiple loci whose phenotypic expression either are affected (plastic) or are not affected (nonplastic) by the environment. Typically, evolution occurred first as genetic differentiation, which was then replaced by the evolution of adaptive plasticity, opposite to the evolutionary trend that is often assumed. Increasing dispersal rates selected for plasticity, if selection occurred before dispersal. If selection occurred after dispersal, the highest plasticity was at intermediate dispersal rates. Temporal variation in the environment occurring after development, but before selection, favored the evolution of plasticity. With dispersal before selection, such temporal variation resulted in hyperplasticity, with a reaction norm much steeper than the optimum. This effect was enhanced with negative temporal autocorrelation and can be interpreted as representing a form of bet hedging. As the number of nonplastic loci increased, plasticity was disfavored due to an increase in the uncertainty of the genomic environment. This effect was reversed with temporal variation. Thus, variation and uncertainty affect whether or not plasticity is favored with different sources of variation—arising from the amount and timing of dispersal, from temporal variation, and even from the genetic architecture underlying the phenotype—having contrasting, interacting, and at times unexpected effects.

## Introduction

We have an apparent paradox. Logic tells us that for organisms living in a heterogeneous environment, natural selection should favor an individual with the ability to change its phenotype so that a trait always expresses optimal values. If a single genotype can express the optimal phenotype in each environment, one might expect it to supplant alternatives, leading to phenotypic diversity underlain by genetic uniformity. Yet in most instances, instead of such ubiquitous phenotypic plasticity, most species differentiate into individuals with a range of fixed phenotypes (Hereford 2009). The apparent exception is polyphenisms, traits with dichotomous phenotypes that differ from the vast majority of traits that vary continuously. We need to explain why adaptive plasticity for continuous traits is much less common than we might expect.

Factors that select against adaptive plasticity can broadly be collected into two categories: costs and limitations (DeWitt et al. 1998). Costs are factors related to plasticity in a trait that reduce the fitness of an individual, even when that trait matches the optimal phenotype across environments. For instance, the developmental machinery required to craft a given phenotype for a range of environments might be costly to maintain. Or, if individuals need to sample the environment in order to assess which environmental conditions are relevant for trait development, there could be costs to such assessments. Limitations are factors that prevent an individual from developing a trait that matches the optimum, even when plasticity per se is cost-free. For instance, imperfect information may make it impossible for a plastic response to create the perfectly optimal phenotype, or developmental constraints may restrict the range of phenotypes that can be achieved. The existence of costs and limitations are two broad answers to the apparent paradox of a lack of ubiquitous adaptive plasticity. However, despite a determined search for costs of plasticity, they rarely have been found (e.g., DeWitt 1995; Scheiner and Berrigan 1998; van Kleunen et al. 2000; Poulton and Winn 2002; Relyea 2002; Callahan et al. 2005, 2008; Weinig et al. 2006; Lind and Johansson 2009; Aubret and Shine 2010; Maherali et al. 2010; see reviews in Van Buskirk and Steiner 2009; Auld et al. 2010). Limitations to plasticity may be more promising as a general explanation for why plasticity is much rarer than is fixed genetic differentiation. In this paper, through the use of simulation models, we explore a range of possible limitations on plasticity and show how they interact to favor or disfavor plasticity.

We need to be clear about the focus of our models: adaptive plasticity. Many traits are plastic without that plasticity necessarily being adaptive (e.g., slower growth or maturation when food is limited). Explanations for nonadaptive trait plasticity lie within the realm of organismal physiology, morphology, and development, and the Theory of Organisms (Scheiner 2010), and are outside the scope of this paper. Instead, we focus on explanations from within the Theory of Evolution, emphasizing the interplay of selection and gene flow in favoring or disfavoring plasticity in heterogeneous environments.

A primary focus of our paper is the dual-edged sword of variation and uncertainty. Selection for plasticity above all else requires a variable environment. If the environment is temporally constant, and selection is stabilizing and frequency independent, one phenotype should outperform all others, so there is no reason for plasticity to occur. Plasticity requires there be variation that alters which phenotype is optimal as the environment changes. But variation always carries within it the seeds of uncertainty. Matching the optimal phenotype to a given environmental state requires an organism to accurately predict what the environment will be when selection occurs. Environmental variation creates the possibility that the environment when an organism's phenotype is determined will differ from the environment during selection. One key source of limitations on plasticity is such uncertainty. Our models expand our understanding of the sources of uncertainty, and allow for more complex evolutionary dynamics than do previous models of plasticity evolution.

This focus on uncertainty is facilitated by recognizing two domains for theories of plasticity evolution. The first domain concerns traits for which the phenotypic value is determined by organismal processes at a rate faster than the rate of change in the selective optimum (e.g., physiology and behavior). In such instances, the evolutionary outcome is largely determined by organismal constraints that include costs of plasticity. For the most part, such explanations should be sought within the Theory of Organisms. As noted above, this paper does not deal with that theory. In contrast, when the rate of trait determination is as slow or slower than the rate of change in the selective optimum, the evolutionary outcome is determined by the interplay of genetic and dynamical population processes whose explanation lies within the Theory of Evolution, our primary focus.

### What we already know

We start by considering what is already known about determinants of plasticity evolution (see review in Berrigan and Scheiner 2004). First, environmental heterogeneity that influences fitness matters. Such heterogeneity can be either spatial or temporal. In either case, plasticity is more favored with greater heterogeneity and higher dispersal rates (e.g., Moran 1992; Gavrilets and Scheiner 1993; Lande 2009). Temporal variation is more likely to favor plasticity than is spatial variation; spatial variation is more likely to favor discrete fixed phenotypes, while temporal variation favors continuous plastic phenotypic variability (Levins 1963; Lynch and Gabriel 1987; Moran 1992). Although typically modeled separately and treated as different phenomena, spatial and temporal heterogeneity in some ways act similarly. From the point of view of a lineage (all of the descendents of an individual), dispersal of a lineage among distinct habitats is similar to temporal variation that occurs among generations at a given location. The two types of heterogeneity differ, however, in that when dispersal is limited, spatial heterogeneity allows for the coexistence of multiple populations with limited interbreeding. Moreover, a given lineage may find itself in multiple local environments in the future, and selection in effect averages over such spatial variability in determining fitness, whereas at a given location, all individuals in a lineage experience the same (changing) environment.

Second, uncertainty matters (Cohen 1968; Orzack 1985; Sasaki and De Jong 1999; Tufto 2000; Sultan and Spencer 2002; Fischer et al. 2011). Environmental variation can lead to uncertainty. By “uncertainty” we mean an information limitation such that the environment experienced by an organism up to the time that the phenotype becomes fixed during development is a less than perfect predictor of the environment at the time that selection occurs. We recognize that often neither development nor selection occurs in a single instant of time, although for convenience, here we model them as such.

Third, life-history matters, specifically in that it influences the likelihood that an organism will experience uncertainty in predicting the environment of selection.De Jong and Behera (2002) showed that for spatial heterogeneity, dispersal has a dual effect on plasticity evolution. On the one hand, it creates the environmental variation experienced by a lineage that is necessary to favor adaptive plasticity. At the same time, however, it can also create uncertainty in an individual's assessment of its likely selective environment, relative to the environment of trait determination.

### What is new

In this paper, we extend and expand on these previous results. We look more deeply at the importance of various factors whose broad patterns are known (e.g., environmental heterogeneity and life-history patterns). In doing so, we show that some generalities are more nuanced than previously recognized, particularly with regard to how the factors interact. Many previous models have considered only one or a few factors; our exploration is more comprehensive, allowing interactions among these factors to emerge.

Of particular note is our exploration of the importance of genetic architecture for the evolution of plasticity. By genetic architecture, we mean the number and type of loci that determine heritable variation in trait phenotype. We define two types of loci that influence the phenotype. The first type consists of loci whose phenotypic expression is not affected by the environment (nonplastic loci). These contrast with loci whose expression is so affected (plastic loci). Selection and evolution of plasticity requires that gene expression be influenced by the environment (i.e., there must be plastic loci). However, a full understanding of why plasticity versus nonplasticity is favored requires that a nonplastic outcome is also possible (i.e., there must be variation at nonplastic loci permitting selection). Recent work confirms the existence of such a mixture of genetic effects in plasticity (e.g., Stratton 1998; Kliebenstein et al. 2002; Promislow 2005; Holeski et al. 2010). Scheiner (1998) showed that genetic architecture—the presence of just plastic loci versus both plastic and nonplastic loci—affects the evolutionary outcome. Those prior results are substantially expanded in this paper.

### Evolutionary outcomes

In this paper, we use an individual-based simulation model to explore the conditions that favor a single plastic genotype that matches the optimal phenotype everywhere, versus multiple genotypes that are fixed for different phenotypes and optimally adapted to local conditions that vary in space or time. We refer to these outcomes as plasticity and genetic differentiation. Of course, these outcomes are not a simple dichotomy. A given evolutionary outcome can include genotypes that both show some plasticity, yet also differ adaptively across a spatial gradient—a mixture of plasticity and local adaptation. For some types of temporal variation in our simulations, there is a third possible outcome: extinction.

## Model Structure

The model consisted of an individual-based simulation implemented in Fortran 77 (Table 1). (The computer code is available from SMS) The metapopulation consisted of a linear array of 50 demes (except for cases where the effect of the number of demes was explored). An environmental gradient was created by varying the optimal value of a single trait (phenotype) in a linear fashion along the array. For 50 demes, the optimum varied from –9.8 to +9.8 arbitrary units at the ends of the gradient, that is, the optimal phenotype in adjacent demes differed by 0.4 units. An individual's phenotype (trait value) was determined by two to 10 unlinked diploid loci: one to five plastic loci and one to five nonplastic loci. The loci in each class contributed additively to the trait, with each allelic value given by a real number. Allelic values at plastic loci were multiplied by an environment-dependent quantity before summing all allelic values. The effect of the environment (*E_i_* for deme *i*) on the phenotypic contribution of each unit plastic allelic value varied in a linear fashion, with a slope of 0.04 units (for 50 demes, *E_i_*= 0.04(*i*–25.5]). The phenotype of each individual was determined at the time of development, and is given by

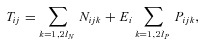
1
where *T_ij_* is the phenotype of the *j*th individual that develops in the *i*th environment (deme), *N_ijk_* is the allelic value of the *k*th nonplastic allele of that individual, *P_ijk_* is the allelic value of the *k*th plastic allele, *l_N_* is the number of nonplastic loci, and *l_P_* is the number of plastic loci. In our model, there is no random component of phenotypic variation. Because both the environmental gradient and the effect of plasticity alleles on the phenotype are linear, perfect adaptation through either genetic differentiation or plasticity is possible.

**Table 1:** Summary of the model parameters.

Fixed parameters
Steepness of the gradient (change in optimum in adjacent demes) = 0.4 units
Strength of selection within demes (σ) = 2 units
Population size = 100 individuals/deme
Number of generations = 10,000 or 20,000
Parameters explored
Dispersal rate
Length of the environmental gradient (50 demes unless otherwise stated)
Number of nonplastic and plastic loci
Life-history pattern: selection before dispersal versus dispersal before selection
Temporal variation: magnitude and autocorrelation

Life-history events occurred in one of two sequences: (1) birth, followed by development (i.e., the phase in the life cycle when the phenotype is determined), then dispersal, selection, and reproduction; or (2) birth, development, selection, dispersal, and then reproduction. For simplicity, we refer to these as “move first” and “select first.” Selection was based on survival with the probability of surviving being a Gaussian function of the difference between an individual's phenotype and the locally optimal phenotype. Fitness (the probability of surviving) was determined as

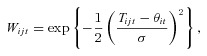
2
where *W_ijt_* is the fitness of the *j*th individual undergoing selection in the *i*th environment in the *t*th generation, *T_ijt_* is the phenotype of that individual, *θ_it_* is the optimal phenotype in that environment at that time, and σ is the strength of selection (a lower value giving stronger selection).

**Figure 1:**
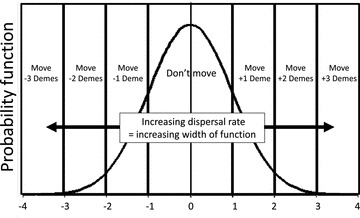
Probability density function for the random variable determining the likelihood that an individual would move between demes and the distance of that movement. Shown is a dispersal probability of 32%. An increase or decrease in that probability is equivalent to increasing or decreasing the width of the function.

The dispersal probability and the distance moved were determined using a zero-mean Gaussian random number, so that the probability of moving and the average distance moved were correlated (Fig. 1). For each individual, the integer part of the random number determined the number of demes moved, with the sign determining the direction of movement. For example, if the Gaussian had unit variance, the probability of dispersal was 32%, because 68% did not disperse at all (i.e., individuals with random numbers between –1 and 1, which for unit variance is within 1 standard deviation of the mean). The fraction of individuals that instead dispersed one deme (half in either direction) was 27% (i.e., those with random numbers with magnitudes between 1 and 2); 5% dispersed two demes (i.e., those with magnitudes between 2 and 3), and so forth. Increasing the dispersal probability was done by increasing the variance of the Gaussian so that more individuals were likely to move and they were likely to move farther. Individuals that would otherwise migrate beyond the end of the gradient migrated to the terminal demes.

Reproduction was accomplished by assembling pairs of individuals within a deme at random with replacement, with each pair producing one offspring, repeating until the carrying capacity of that deme was reached (in our simulations, this was 100 individuals per deme). This procedure assumes soft selection, in that local population size was determined independently of the outcome of selection, and also assumes that the spatial scale of reproduction and mating matches that of density dependence and the grain of the selective environment. (We will relax the assumption of soft selection in a future contribution.)

Each simulation was initialized with 100 individuals being “born” in each deme. Unless otherwise indicated, for each individual in the initial generation, allelic values (for both plastic and nonplastic loci) were chosen independently from the values −2, −1, 0, 1, and 2, with each value being equally likely (even though initial values are discrete, due to mutation, allelic values are continuous variables after the initial generation; see below). Starting a simulation with no genetic variation (i.e., all alleles at value 0) had no effect on the final equilibrium, and simply slowed the time to reach equilibrium as genetic variation had to build up through mutation. Changing the starting allelic values also had no effect on the final equilibrium, that is, there was no evidence of multiple evolutionary equilibria at a given parameter combination.

**Figure 2:**
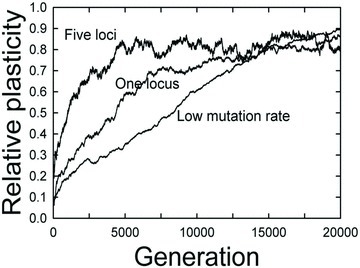
An example showing the effect of mutation rate and the number of plastic loci on the number of generations to reach equilibrium. A value of 1.0 for relative plasticity indicates a pure plasticity outcome. The figure shows three typical runs. For all, there were three nonplastic loci. The labels “five loci” and “one locus” indicate the number of plastic loci. Those runs both had high mutation rates (10%). The “low mutation rate” run had one plastic locus and a mutation rate one-tenth that of the other runs (1%). For these simulation, the dispersal rate was 32%, dispersal occurred before selection, and the simulations were begun with genetic variation.

When new offspring were generated, each allele mutated with a probability of 10%. When a mutation occurred, the allelic value was changed by adding a Gaussian deviate with a mean of zero and a standard deviation of 0.01 units to the previous allelic value (i.e., this is an infinite-alleles model). Decreasing the mutation rate did not change the mean values at equilibrium (Fig. 2), although it did decrease the standing genetic variation maintained through mutation–selection–drift balance and increased the time needed to reach equilibrium.

Temporal variation in selection occurred within a deme by varying the optimal phenotype just before selection occurred, once per generation. To allow for temporal autocorrelation, the optimal phenotype was calculated using the recursion

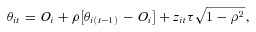
3
where *θ_it_* is the optimal phenotype in the *i*th environment in generation *t*, *O_i_* is the mean or fixed optimal phenotype in the *i*th environment (a linear function of *i*), τ is the standard deviation of environmental variation, ρ is the temporal autocorrelation coefficient, and *z_it_* is a sequence of independent zero-mean, unit-variance Gaussian random deviates chosen independently for each deme. For simulations without temporal variation, τ= 0, and for uncorrelated temporal variation, ρ= 0. For results with temporal variation, the standard deviation of environmental noise (τ) is given as a percentage of the difference in the optima at the two ends of the gradient. The autocorrelation (ρ) was varied from –75% to 75%. The environment at the time of development (*E_i_*) did not vary among generations.

All simulations were run for 10,000 or 20,000 generations, depending on the parameter combination, to ensure that the equilibrium point (the point after which all calculated quantities showed no directional trend) was reached. The number of generations required to reach equilibrium depended on the number of loci, and was around 7500 for 10 loci and 15,000 for two loci, mainly due to the faster build-up of genetic variation with more loci. Figure 2 shows examples of the approach to equilibrium for eight loci (three nonplastic and five plastic) and four loci (three nonplastic and one plastic). Each parameter combination was run 20 times, and the results described below depict mean outcomes. If the meta-population went extinct, additional realizations were run until 20 successful replications were achieved; for some parameter combinations (see results), the extinction probability was 100% (i.e., no successful replications in 1000 runs). Reported outcomes were averaged over successful replications only.

The reaction norm is a mathematical function describing how the phenotypic expression of a given genotype varies among environments. A linear reaction norm is best described by the slope of the function. In our model, the slope of the reaction norm is the product of the slope of *E_i_* and the sum of the values of the plasticity alleles (i.e., the right-hand term in eq. 1). For these simulations, as the slope of *E_i_* was identical, the final outcome was measured as the average across all demes of the sum of the values of the plasticity alleles for each individual. That is, 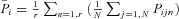, where 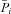 is the mean plasticity of the *i*th deme over all *r* runs, *N*= 100 is the number of individuals per deme, and *P_ijn_* is the sum of the values of the plasticity alleles of the *j*th individual developing in the *i*th deme in the *n*th run. The overall 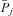 mean plasticity is the average of 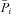 across demes, and is given by 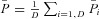, where *D* is the number of demes. (The order of averaging, over runs within demes first or over demes within runs first, does not affect the final average because the number of demes is the same for all runs. Mean plasticity was calculated at each generation.) The average plasticity was standardized to the optimal reaction norm (giving the relative plasticity) so that a pure plasticity outcome would have a value of 1 and a pure differentiation outcome would have a value of 0. Values outside this range were possible; that is, it was possible to achieve a reaction norm with a slope steeper than the optimal value (>1) or in the opposite direction from the optimal value (<0). The average reaction norm differed among demes with lower absolute slope values in the middle of the metapopulation (Scheiner 1998; Fig. 1) because of the way that the optimal phenotype changed from negative to positive arbitrary units along the gradient. However, for our purposes, the global average reaction norm provides a sufficient summary of the overall pattern of adaptation.

## Results

### The time course of evolution

**Figure 3:**
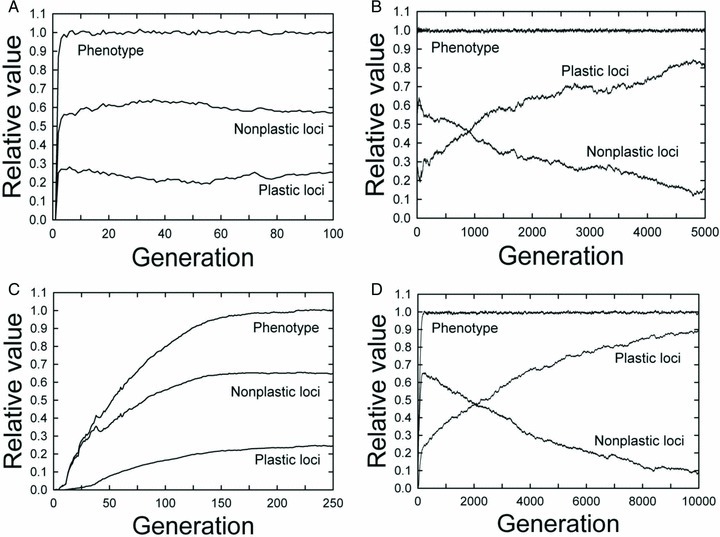
Typical time courses of evolution with (A, B) and without (C, D) genetic variation in the initial generation, shown for the initial generations (A, C) and to equilibrium (B, D). Shown are the relative values for the total phenotype, and sum of allelic values for the nonplastic and plastic loci. All values are averaged across all demes. “Phenotype” indicates the mean phenotype, standardized so that a value of 1.0 indicates that the mean is at the optimum in all demes. It was calculated as the slope of the mean phenotypes in each deme divided by the slope of the optimal phenotype. “Nonplastic loci” and “Plastic loci” are the sum of the allelic values, standardized to the optima (for plastic loci, this is the same as relative plasticity). A value of 1.0 for “Nonplastic loci” would indicate that the variation among demes was entirely due to genetic differentiation, calculated as the slope of the mean allelic sum in each deme divided by the slope of the optimal phenotype. A value of 1.0 for “Plastic loci” would indicate that the metapopulation had achieved the optimal reaction norm. For these simulations, the numbers of nonplastic and plastic loci were five each, the dispersal rate was 32%, and dispersal occurred before selection.

All simulations started with a mean phenotype of 0 units and a mean reaction norm of 0 in all demes. Typical time courses of evolution for selection after dispersal (move first) scenarios are shown in Figure 3. When genetic variation was present initially, within a few generations the metapopulation achieved the selective optimal phenotype in all demes, primarily through selection on the nonplastic loci, that is, through genetic differentiation among populations (local adaptation along the gradient; Fig. 3A and B). After an initial response of the plastic loci toward the optimal reaction norm, plasticity reached a plateau at a low level until approximately generation 50. Thereafter, plasticity was selected for until equilibrium was reached at approximately generation 5000. When the same parameter combination was started with no genetic variation (Fig. 3C and D), the same overall pattern of response occurred, except that the duration of the initial response and the time to equilibrium was delayed as mutational variation was generated. This pattern of genetic differentiation arising first, then being displaced (often over a long time scale) by adaptive plasticity, is opposite to the pattern that is usually assumed (Baldwin 1896; Schlichting 2004). The parameter combination used in these examples favored plasticity, as was typical for the move first scenarios (see below).

### External uncertainty—dispersal rate

**Figure 4:**
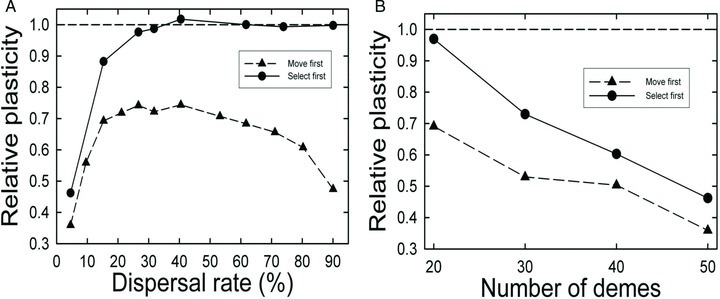
(A) The interaction of dispersal rate and life-history pattern on the evolution of phenotypic plasticity. A relative plasticity value of 1.0 indicates a pure plasticity outcome. (B) The effect of gradient length (number of demes) on the evolution of phenotypic plasticity. The difference in the selective optima between adjacent demes was the same for all simulations, so that increasing the number of demes changed the length of the gradient, not the optimal reaction norm. The dispersal rate was 4.6%.

As expected, the dispersal rate determines the extent to which plasticity is favored over genetic differentiation. However, this effect depends strongly on the life-history pattern (Fig. 4A). When selection occurs prior to dispersal (select first), so that development and selection happen in the same environment, moderate to high dispersal rates (15% and above) strongly favor plasticity. In this instance, dispersal creates variation in the selection experienced by a lineage from one generation to the next, favoring those genotypes that can adjust their phenotype to that variation.

In contrast, selection after dispersal (move first) implies that development and selection occur in different environments. This ordering of life-history events favors plasticity most at intermediate rates of dispersal (20–40%). In this instance, because dispersal creates variation in the environments experienced within an individual's life span, that variation also creates uncertainty of matching the optimal phenotype. At all dispersal rates, plasticity is less favored in the move first life-history pattern, and dispersal rates above 50% begin to further disfavor plasticity for the move first scenario. In our simulations, dispersal rate was coupled with the likely distance moved, magnifying at high dispersal rates the lack of phenotype matching, and thus the uncertainty of assessing environmental states. However, even at high dispersal rates (high uncertainty), some plasticity is favored.

For organisms with a plastic genotype that produces a single, fixed phenotype within a given life span (as we are assuming), environmental heterogeneity changes from variable but predictable (for select first) to variable and uncertain (for move first), depending on when during the life cycle events occur. Predictable variation selects for plasticity and uncertainty selects against plasticity. The result is a balance between these processes so that the move first life history leads to a mixed outcome with both plasticity and local adaptation. Therefore, the evolutionary outcome depends on the life-history pattern.

The different effects of environmental heterogeneity can be seen by varying the length of the environmental gradient (Fig. 4B). For both life-history patterns, at the lowest dispersal rate (4.6%), as the number of demes increases, plasticity is less favored. This effect is much smaller at a dispersal rate of 15% and completely disappears at a dispersal rate of 26% (results not shown). At very low dispersal rates, an increase in the number of demes tips the balance toward genetic differentiation, likely through a simple isolation-by-distance effect. Increasing the dispersal rate eliminates this effect because a given lineage is more likely to experience more environmental variation. So the rate of dispersal interacts with the size of the evolutionary arena in determining how much plasticity is favored.

### External uncertainty—temporal heterogeneity

**Figure 5:**
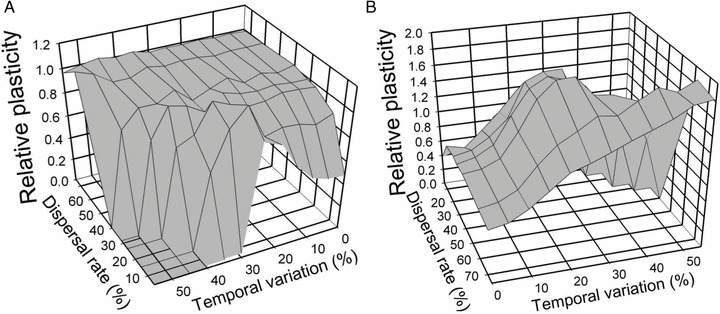
The interaction of dispersal rate and the standard deviation of the local phenotypic optima (temporally varying with no autocorrelation) on the evolution of phenotypic plasticity. Temporal variation is scaled as a percentage of the length of the environmental gradient. All simulations had five nonplastic and plastic loci. (A) Selection before dispersal (select first). (B) Dispersal before selection (move first). Low dispersal and high temporal variation led to 100% extinction; for these parameter combinations, relative plasticity is shown as 0 (front left in A, back right in B). Note that horizontal axes are reversed in the two graphs; if the right figure were to be rotated 180^o^, you would see a “cliff” similar to that in the front left-hand corner of A (where parameters sets lead to 100% extinction).

Temporal variation in the optimal phenotype interacts with both dispersal rate and life-history pattern and changes the effect of uncertainty. When there is no temporal autocorrelation, very high temporal variation coupled with low dispersal rates mostly results in population extinction (shown as values of relative plasticity of 0 in Fig. 5). When selection occurs before dispersal (select first), high dispersal rates always select for the optimal reaction norm regardless of the amount of temporal variation (Fig. 5A), indicating that selection for plasticity due to environmental heterogeneity overcomes selection against plasticity due to uncertainty. At very low dispersal rates (<20%), increasing amounts of temporal variation favors plasticity up to variation approximately equal to 25% of the length of the environmental gradient; above that the metapopulation is most likely to go extinct.

**Figure 6:**
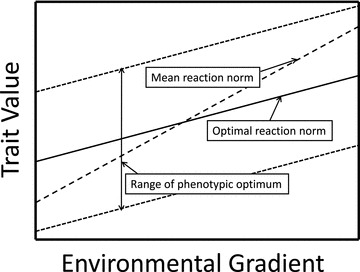
When there is within-deme temporal variation in the environment, the mean phenotypic optimum across demes no longer predicts the optimal reaction norm. The optimal reaction norm will be steeper than this mean (e.g., high dispersal and temporal variation in Fig. 5B) because of the increase in the total range of environmental variation across the metapopulation. The exact difference between the mean phenotypic optimum and the optimal reaction norm will depend on the frequency distribution of the temporal variation.

In contrast, dispersal before selection (move first) can result in a mean reaction norm that is substantially steeper than the optimal reaction norm, up to nearly twice as steep for combinations of very high temporal variation and dispersal rate (Fig. 5B; axes reversed relative to A). The optimal reaction norm is determined by the range of the environmental variation. Temporal variation raises the selected phenotype relative to the optimum at one end of the gradient and lowers it at the other (Fig. 6). As with no temporal variation, selection for plasticity is maximized at an intermediate dispersal rate, but the peak is found at increasingly greater dispersal rates as temporal variation increases. Thus, even as increasing temporal variation makes phenotype matching less certain for individuals that do not disperse, plasticity paradoxically becomes more favored. We resolve this paradox in the Discussion.

**Figure 7:**
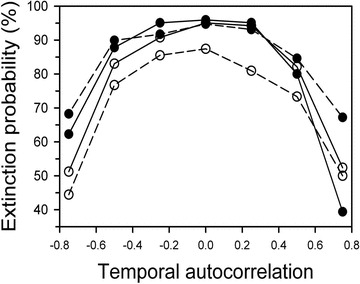
The effects of temporal variation of local phenotypic optima on the probability of the metapopulation going extinct. Open symbols = move first; closed symbols = select first; dashed lines = dispersal rate of 15% and a standard deviation of temporal variation 35% the length of the environmental gradient; solid lines = dispersal rate of 32% and a standard deviation of variation of 45%. All simulations had five nonplastic and five plastic loci.

**Figure 8:**
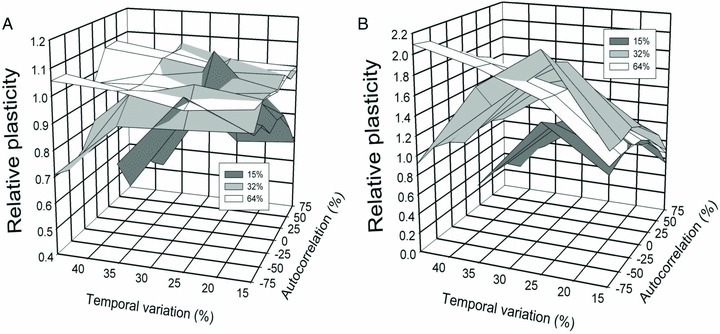
The interaction of temporal variation and autocorrelation on the evolution of plasticity for different dispersal rates (15%, 32%, 64%). Temporal variation is scaled as a percentage of the length of the environmental gradient. A value of 1 indicates that the metapopulation converged to a pure plasticity outcome; values less than that indicate that the average reaction norm had a slope intermediate between the two pure outcomes. Values greater than 1 indicate that the average reaction norm was steeper than the optimal value. All simulations had five nonplastic and plastic loci. (A) Selection before dispersal (select first). (B) Dispersal before selection (move first). No values are shown for parameter combinations of a 15% dispersal rate and 45% temporal variation because the metapopulation went extinct.

Temporal autocorrelation creates further complications. First, given that there is dispersal along the gradient, the probability of extinction is lower with temporal autocorrelation, whether that autocorrelation is positive or negative (Fig. 7). When selection occurs before dispersal (select first), high dispersal rates select for the optimal reaction norm regardless of the amount of temporal variation or the amount and sign of temporal autocorrelation (Fig. 8A). At intermediate and low dispersal rates, plasticity is less favored at high amounts of temporal variation and autocorrelation (both positive and negative).

By contrast, when dispersal occurs before selection (move first), dispersal rate interacts with autocorrelation to influence selection on plasticity (Fig. 8B). When dispersal rates are low, plasticity is most favored at intermediate values of temporal variation and autocorrelation has little effect. When dispersal rates are high, plasticity is favored when temporal variation is high and strongly negatively autocorrelated. In those instances, maximal plasticity is twice as large as the optimal mean reaction norm. For high dispersal rates and positive autocorrelation, maximal plasticity is much closer to the optimal reaction norm. Negative autocorrelation increases variation in phenotype matching and makes it highly likely that the environment of selection will be very different from one generation to the next, resulting in the evolution of extreme plasticity much greater than the optimal value. In contrast, positive autocorrelation decreases both variation among generations and uncertainty, thereby selecting for the optimal reaction norm.

### Internal uncertainty—genetic architecture

**Figure 9:**
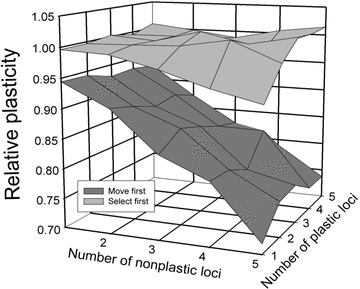
The effect of genetic architecture on the evolution of phenotypic plasticity. A value of 1 indicates that the metapopulation converged to a pure plasticity outcome; values less than that indicate that the average reaction norm had a slope intermediate between the two pure outcomes. For these simulations, the dispersal rate was 32%.

Genetic architecture (i.e., the number of loci) also affects selection for or against plasticity, but this effect depends on life history (Fig. 9). When selection occurs before dispersal (select first), genetic architecture has almost no effect, except for a minor effect when the number of plastic loci is 1. In contrast, when dispersal occurs before selection (move first), increasing the number of nonplastic loci selects against plasticity; by contrast, the number of plastic loci has no effect.

**Figure 10:**
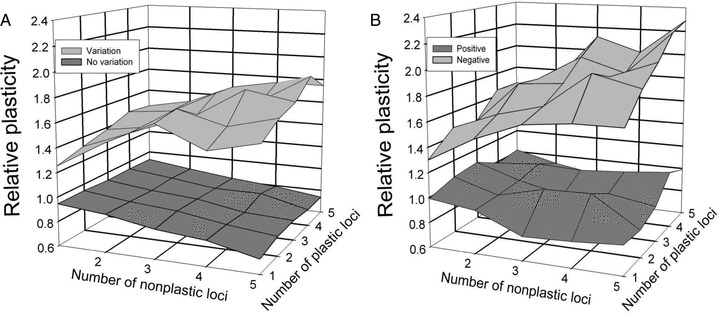
The interaction of temporal variation with genetic architecture on the evolution of phenotypic plasticity when dispersal occurs before selection (move first). A value of 1 indicates that the metapopulation converged to a pure plasticity outcome; values less than that indicate that the average reaction norm had a slope intermediate between the two pure outcomes. Values greater than 1 indicate that the average reaction norm was steeper than the optimal value. For these simulations, the dispersal rate was 32%. The standard deviation of the temporal variation was 25% of the length of the environmental gradient. (A) No temporal variation contrasted with uncorrelated temporal variation. (B) Positive temporal autocorrelation contrasted with negative autocorrelation; the magnitude of the autocorrelation was ±75%.

These effects interact with those of temporal variation, such that the effect of genetic architecture reverses as temporal variation increases (Fig. 10). When there is no autocorrelation (Fig. 10A), as the number of both nonplastic and plastic loci increases, plasticity is more favored, resulting in maximal plasticity nearly twice as large as the optimal mean reaction norm. With negatively autocorrelated environments, this effect is even larger (Fig. 10B). With positive autocorrelation, reaction norms are similar to conditions with no temporal variation, although there is a suggestion that the number of plastic loci matters when the number of nonplastic loci is large. Below, we suggest that these effects arise because genetic architecture itself leads to a kind of uncertainty in the selective environment.

## Discussion

### Variation versus uncertainty

Variation and uncertainty affect whether or not plasticity is favored, with different sources of variation—amount and timing of dispersal, temporal variation, and genetic architecture—having contrasting, interacting, and at times unexpected effects. Variation comes about by changes in the selective optimum experienced by an individual (within a generation) or a lineage (across generations). Uncertainty is caused by differences between the environment at the moment when the phenotype is determined and at the moment when selection occurs. Variation generally selects for plasticity (e.g., high dispersal rates after selection), while uncertainty generally selects against plasticity (e.g., high dispersal rates prior to selection) (Fig. 4).

Whether or not a particular form of variation is uncertain is context dependent. For temporal variation and selection prior to dispersal (Fig. 8A), when dispersal rates are low, variation adds uncertainty and selects against plasticity. However, once dispersal rates are high (i.e., among-generation variation is large), additional temporal variation no longer creates additional uncertainty. A given lineage is nearly certain to experience variation whether or not dispersal occurs. Temporal autocorrelation has little effect in this situation.

In contrast, when dispersal occurs before selection and dispersal rates are high, high temporal variation overcomes uncertainty. Even more dramatically, negative autocorrelation acts synergistically to select for extreme plasticity. That is, high dispersal rates plus high temporal variation combined with high negative autocorrelation makes change extremely likely, thus favoring plasticity for this life history.

These interactions between the amount and timing of dispersal and the amount and pattern of temporal variation are not intuitively obvious. It is obvious why selection before dispersal with no temporal variation should nearly always select for the optimal reaction norm (Fig. 5A), a result consistent with previous models (Via and Lande 1985; Moran 1992; Jablonka et al. 1995; De Jong 1999; Sasaki and De Jong 1999; De Jong and Behera 2002). What is not obvious is why temporal variation has little effect (Fig. 5A), as such variation creates uncertainty that should select against plasticity. It may be that the strength of selection within demes is low, and that the balance between variation and uncertainty might be altered if the strength of selection were increased.

### Plasticity as bet-hedging

Dispersal before selection leads to much more complex patterns. We unexpectedly found that temporal and spatial variation act synergistically to favor plasticity (Fig. 5B). One's first intuition is that within-generation temporal variation (i.e., changes in the environment between development and selection even if no dispersal occurs) should add uncertainty and disfavor plasticity. Instead, increasing rates of dispersal and greater amounts of temporal variation together favor quite extreme plasticity. This effect can be understood if one considers selection on a lineage, rather than an individual. When dispersal rates are high, the members of a lineage quickly spread across the entire metapopulation, likely experiencing a wide range of environments. Temporal variation simply enhances this effect, pumping up the amount of variation to which that lineage is exposed.

In this case, rather than plasticity per se being adaptive, it acts as a form of bet-hedging by producing increased phenotypic variation among offspring. If we think of fitness as the number of grandchildren produced by an individual, and recall that fitness is averaged over all offspring, in conditions with high temporal variation and high dispersal rates, a plastic lineage is likely to have at least some of its members enjoy high fitness in some demes. Negative autocorrelation further enhances this effect because even individuals that do not disperse are likely to experience a very different environment from that experienced either during selection in the previous generation or during development in the current generation. As far as we are aware, these interactions of temporal and spatial variation and these effects of temporal autocorrelation have not been previously explored.

Previous models of the evolution of bet-hedging or adaptive coin flipping have considered the evolution of developmental noise—nonenvironmentally determined phenotypic variation (e.g., Kaplan and Cooper 1984; Simons and Johnston 1997; Einum and Fleming 2004; Acar et al. 2008; Donaldson–Matasci et al. 2008; Rees et al. 2009) (see review in Philippi and Seger 1989). In our model, there is no developmental noise. The only within-genotype phenotypic variation is from plasticity. Our results imply that some instances of seemingly nonadaptive plasticity, especially plasticity that causes phenotypes beyond the optimum, may result from selection for bet-hedging. In assessing amounts of trait plasticity, hyperplasticity might be an indication of bet-hedging. When trying to determine if trait plasticity is adaptive or selection for bet-hedging, it is necessary to go beyond the average environment at a site and look at temporal variation and its autocorrelation, and how sites with different environmental means and histories are coupled by dispersal.

### Context-dependent selection

The effects of gene number on the evolution of plasticity have not been previously recognized. With regard to the evolution of plasticity, the “environment” has always implicitly been the external environment (Berrigan and Scheiner 2004). Our results point to the fact that the internal environment—the rest of the genome—can be just as important. That selection on plasticity was affected by the number of nonplastic loci was completely unexpected. Our a priori expectation was that the plasticity of the metapopulation either would not vary with the number of loci, or would depend only on the number of plastic loci. Instead, we found (Figs. 9 and 10) that the evolution of plasticity was sensitive to the number of nonplastic loci determining trait values.

Our result can be explained by recognizing that for a plastic allele, the optimal phenotypic expression in a given environment depends on the expression of the other alleles in the genome. Under free recombination (i.e., no linkage), as the number of loci increases, the possible number of genomic environments experienced by an allele increases geometrically. A plastic allele experiencing a particular gene combination in one generation may experience a very different gene combination in the next generation. Increasing the number of nonplastic loci has the effect of increasing uncertainty in the fitness environment, analogous to the effects of increasing the dispersal rate across a gradient or the amplitude of temporal variation. The unpredictability of the genomic environment can thus be just as strong a force of selection on the evolution of plasticity as is the unpredictability of the external environment.

These results reinforce the notion that genotype– environment interaction (G × E) effects should be treated as a form of cross-environment epistasis (Scheiner and Lyman 1991).Wolf et al. (2004) posit a model of context-dependent evolution for which that context can be both the environment and the rest of the genome. That model was based on an analysis of within-generation fitness in a single panmictic population. However, we know that selection on epistatic interactions is enhanced when there is population substructuring (Wright 1969). Our results point to the importance of considering the net selection on a gene lineage across all demes within a metapopulation and across generations, and emphasize the fact that the rest of the genome is a key dimension of the selective milieu acting on any particular locus.

### Genomic analyses

These effects of genomic architecture have important consequences for the way that genomic analyses are designed. Much more genomic information, both gene expression profiles and Quantitative Trait Loci (QTL) numbers, is needed on the environmentally conditioned effects of genes on phenotype. While some data exist (e.g., Gurganus et al. 1998; Stratton 1998; Callahan et al. 2005; Hausmann et al. 2005; Promislow 2005; Ellers et al. 2008), it is rarely collected in the context of a species’ ecology (e.g., Wu 1998; Weinig et al. 2003a, b; Aubret and Shine 2010; Holeski et al. 2010).

Were such data to be collected, our results make several predictions: (1) Comparisons among populations within a species differing in amounts of adaptive trait plasticity will find more nonplastic loci with standing variation in meta-populations with less plasticity. (2) The number of plastic and nonplastic loci will be negatively correlated among traits or among metapopulations for a given trait. (3) Standing variation for plastic loci will be revealed if individuals are raised under environmental conditions that they typically do not encounter in nature. That is, under typical conditions, selection against plasticity will tend to select for reaction norms with slopes of 0. (4) Because linkage decreases the amount of among-generation genetic variation, adaptive trait plasticity will tend to be associated with tight linkage among non-plastic loci, and possibly between plastic and nonplastic loci. We emphasize that these predictions depend on the life-history pattern of the species (Fig. 9) and the amount and pattern of temporal variation (Fig. 10). Blanket predictions are unwarranted.

### Species invasions

The effect of gene number on plasticity evolution provides novel insights into and predictions about species invasions. Invasive species typically go through a bottleneck during initial colonization. If that bottleneck eliminates genetic variation of nonplastic loci, colonizing populations are freed to evolve greater amounts of trait plasticity, which could facilitate invasion of additional novel habitats. This evolution of plasticity after colonization could help explain the lag period between colonization and invasive spread (Gurevitch et al. 2011). We predict that comparisons of invasive species between nonnative and native ranges will find that nonnative populations have greater trait plasticity and fewer nonplastic loci with standing variation. Our hypothesis concerning the process of species invasion provides a specific mechanism for the previously suggested hypothesis of plasticity evolution after invasion (Richards et al. 2006). We caution that our prediction holds only for some life-history patterns. The process of species invasion is complex, and it is unclear how the effect that we posit might interact with others. A recent meta-analysis found that invasive species are, on average, more plastic than native congeners, but that plasticity was not associated with greater fitness (Davidson et al. 2011). That analysis however did not distinguish between preexisting or newly evolved plasticity, so it is unclear whether the results are consistent with our predictions. A full exploration of these predictions will require integrating our proposed mechanism into the larger conceptual framework of the theory of invasion (Gurevitch et al. 2011).

### Extinction

The principal aim of our study was to examine how, within a species distributed along a gradient, different factors affect the relative partitioning of adaptive phenotypic variation into plastic and nonplastic contributions. But plasticity has important consequences in itself, for instance in influencing extinction risks of species in harsh or changing environments. It makes intuitive sense that adaptive plasticity, by permitting individuals to (partially) match their phenotype to local conditions, should enhance a population's persistence. This intuition has been quantified and refined in several recent contributions. Lande and collaborators (Lande 2009; Chevin and Lande 2010; Chevin et al. 2010) extended the results of Gomulkiewicz and Holt (1995) by examining how the evolution of plasticity can permit the persistence of a population in a changing environment. Plasticity reduced the initial rate of decline in abundance of a population suddenly exposed to an abrupt change in the environment. This greater abundance then makes it more likely that adaptive evolution can sufficiently increase mean fitness to rescue the population from its impending extinction.

However, Reed et al. (2010) caution that plasticity can also hamper persistence, if the environmental cues that determine development are decoupled from the environment of selection; their model most closely resembles our “move first” scenario or instances with high within-generation temporal variation. In our model, our assumption of soft selection means that in most circumstances population persistence is assured. Extinction can nonetheless arise if the dispersal rate is low and there is large-scale temporal variation. Extinction risks were greatest for both life-history patterns when the temporal autocorrelation and dispersal rates were low; for the “move first” life-history pattern, this risk was somewhat lower when the range of temporal variation was somewhat lower (Fig. 7). These results contrast with the conclusions of Reed et al. (2010). However, theirs is a nonevolutionary model with a fixed amount of plasticity. In our simulations, the initial evolutionary response is genetic differentiation (Fig. 3), and many of the extinctions occurred rapidly, before there was an opportunity for the evolution of high plasticity. Parameter combinations with a lower risk of extinction tended to evolve toward greater plasticity (Fig. 8). We caution that these results measure the plasticity only of the surviving metapopulations; in these simulations, we did not measure the amount of plasticity at the time of extinction. In future contributions involving hard selection, we intend to examine in more detail how evolution of plasticity and local adaptation influence species persistence in temporally and spatially varying environments, including at range margins.

### The anti-Baldwin effect

Over a century ago, Baldwin (1896) posited that the process of divergence and adaptation to a range of environments could be enhanced by phenotypic plasticity. Plasticity would allow a species to initially inhabit a wider range of environments, following which the populations in each of those environments would become further adapted through genetic differentiation with a subsequent diminution of their plasticity. This idea was encapsulated into Waddington's (1953) ideas of canalization, and more recently has been proposed as a source of phenotypic novelty (West-Eberhard 2003, 2005) and as a prelude to speciation (Schlichting 2004). The recent models of Lande (2009) and Thibert-Plante and Hendry (2011) demonstrate this process. However, early on Wright (1931) pointed out that plasticity could also inhibit genetic divergence. If a species is plastic such that its phenotype matches the optimum in all environments, there would be no impetus for further evolution.

Our model is the first to demonstrate an anti-Baldwin effect, namely the displacement of a set of genetically differentiated genotypes by a plastic genotype (Fig. 3). This dynamic may be widespread among models. Most models of plasticity evolution are either analytical or, if computational, examine just equilibrial behavior and not transient dynamics. Displacement of differentiation by plasticity might be found, once looked for. Even in our modeling efforts, we mostly ignored transient dynamics once we confirmed that starting conditions did not affect the outcome, except to insure that the simulations were run for a sufficient time to reach equilibrium.

Even if equilibrial conditions favor plasticity, transient dynamics can be extremely important for empirical systems where conditions are rarely stable for hundreds or thousands of generations. Other factors can affect transient dynamics, in particular genetic linkage. The results presented here were for unlinked loci. When we examined the effects of linkage, we found that they had no effect, except for the case of complete linkage, which showed some evidence for transient effects lasting a few tens of generations while mutational input degraded genetic correlations. These transient effects did not change the equilibrial outcome (results not shown). However, the mutation rate used in our simulations was high; it was chosen to make the simulations reach equilibrium in a reasonable amount of time (Fig. 2). Lower mutation rates likely would magnify the importance of transient dynamics.

### Left undone

A conspicuous difference between our model for the evolution of plasticity and others is its spatial structure. All other models of plasticity evolution (except De Jong 1995; Scheiner 1998) that have spatial variation consist of just two demes or two environmental states (see review in Berrigan and Scheiner 2004). Our linear spatial structure and correlation between dispersal rate and distance traveled create a positive correlation between the range of environmental variation an individual experiences during its lifetime and the variation a lineage experiences over generations. Temporal variation then interacts with these effects. It is unclear whether the relative importance of dispersal and temporal variation would change if distance traveled was uncoupled from dispersal rate (the fraction of individuals leaving their natal environments). Nor is it clear if the behavior of our model would change if there were only two demes. Further modeling is needed.

The mode of selection—hard versus soft—affects the evolution of phenotypic plasticity (Van Tienderen 1991). Our current efforts only consider soft selection. However, in many circumstances, selection will be hard. Recent theoretical literature on adaptive evolution in source–sink environments (e.g., Kawecki 1995; Holt 1996a; Ronce and Kirkpatrick 2001; Kawecki and Holt 2002; Alleaume-Benharira et al. 2006) and on evolution at range margins has explicitly focused on the feedback between demography and adaptation, to understand how evolution can alter absolute fitness so that populations adapt and persist in environments in which they initially were headed toward extinction. In many of these models, natural selection tends to be biased against adaptation in low-density, sink environments, and in the populations at range margins. This bias against adaptation occurs because of how selection averages over populations that vary in abundance (Holt and Gaines 1992; Kawecki 1995; Holt 1996b; Ronce and Kirkpatrick 2001; Cohen 2005). It also reflects how immigration can depress fitness, given density dependence (Holt 1996a), and how genetic drift erodes genetic variation in marginal populations (Ronce and Kirkpatrick 2001). Finally, this bias occurs because in outcrossing sexual species, gene flow can impose a high migrational load in low-density populations (Kirkpatrick and Barton 1997). The evolution of plasticity can potentially alter all these effects, at least quantitatively, and possibly qualitatively.

### Adaptive plasticity

We began by noting that adaptive plasticity appears to be rare. Yet in our simulations, for most parameter combinations, plasticity was favored. We have several explanations for this discrepancy. First, adaptive plasticity may be more common than appears. Our initial statement is based on the analysis of Hereford (2009) that found evidence that adaptive genetic differentiation was very common. But that analysis does not preclude adaptation due to partial differentiation combined with partial plasticity. Such a mixed strategy has been found (e.g., Scheiner and Teeri 1986), and there are many examples of adaptive plasticity (DeWitt and Scheiner 2004). However, such evidence is anecdotal in the sense that it is simply an accounting of single studies, many of which were done on systems where adaptive plasticity was suspected, resulting in an ascertainment bias. Needed is a meta-analysis of adaptive plasticity similar to the analysis of Hereford. Second, some parameter combinations in our models predict the evolution of hyperplasticity. An empirical study would conclude that such plasticity is not adaptive because of the mismatch between the realized and optimal reaction norms. This would be mistaken, because adaptation has to be viewed from the perspective of genetic lineages, which average over all the conditions experienced by the individuals that carry the genes. Third, as indicated in the previous section, our current simulations are limited to soft selection. Fourth, even with all of the variables and parameter combinations that we explored, there is much more unexplored parameter space. In particular, these simulations did not include costs of plasticity; a future contribution will examine this factor and our preliminary results show that as is intuitively reasonable, such costs can substantially reduce selection for plasticity. Finally, we primarily examined equilibrial outcomes. As we have shown (Figs. 2 and 3), it can take hundreds of generations for plasticity to come to high frequency, even when favored. So a lack of adaptive plasticity in empirical systems may be due to the world not being at equilibrium. Of course, such an explanation is rather unsatisfying because it is a hypothesis that is very difficult to disprove.

### Model testing

Models in evolutionary biology have a curious history. Very few models are directly tested. By directly tested, we mean an instance where a specific model makes a precise, quantitative prediction that is compared to data from an experimental or natural system. Instead, models typically serve as heuristic guides to understanding or provide qualitative predictions. This has been especially true in our understanding of the evolution of plasticity. Formal models of plasticity evolution began with Via and Lande (1985), although statements about expectations of plasticity evolution go back as far as Baldwin (1896) and Wright (1931). Over the past 25 years, many models have been published (see review in Berrigan and Scheiner 2004), although the pace has substantially diminished in the past 10 years.

Despite this sizable set of models, none have been directly tested, as far as we are aware. That is not to say that general, qualitative predictions have not been tested. These qualitative predictions have been long known (DeWitt et al. 1998) and have held up (DeWitt and Scheiner 2004). For example, higher dispersal rates are associated with greater plasticity (Hollander 2008). Even so, as shown by our results and those of others (De Jong and Behera 2002), details matter and can lead to very different predictions.

This complexity of predictions makes testing these models particularly difficult. There are extensive logistical difficulties. Because plasticity evolution requires populations spread over multiple environments, examination of natural populations for congruence with model predictions is hindered by the necessity for replicate metapopulations. It is difficult enough finding multiple populations that differ in only a single key factor; that problem is magnified when scaled up to whole metapopulations. Although experimental tests solve the problem of uncontrolled variation, such tests require complex experimental designs (e.g., Scheiner and Lyman 1991). The complexity of our results with its multiway interactions of life-history pattern, environmental variation, and genetic architecture reinforces this somewhat gloomy outlook. We have long known that testing models of plasticity evolution is hard, likely accounting for the paucity of any such tests. A notable exception is the recent study of Lind et al. (2011) that examined multiple metapopulations of a species of frog. These authors found a positive relationship between dispersal rates and plasticity of tadpole development time, congruent with the predictions of our “select first” model.

So, how should we treat our results? As with most evolutionary models, they serve best as a heuristic tool for informing our thinking about general evolutionary processes. They provide general guidelines for what to expect in system behavior. Rather than trying to test model predictions, model robustness can be examined by focusing on model assumptions and parameter values and by comparing qualitative predictions among models that use very different approaches. For example, our model predictions concerning the effects of life-history pattern (selection before or after dispersal) are congruent with others (De Jong and Behera 2002). Such teasing out of broad, qualitative patterns and the understanding that they give us of the evolutionary process may be the best that models such as these can provide.
